# Role of endometrial blood flow assessment with color Doppler energy in predicting pregnancy outcome of IVF-ET cycles

**DOI:** 10.1186/1477-7827-8-122

**Published:** 2010-10-18

**Authors:** Lina Wang, Jie Qiao, Rong Li, Xiumei Zhen, Zhaohui Liu

**Affiliations:** 1 Reproductive Medicine Center, Peking University Third Hospital, Beijing 100083, P. R. China

## Abstract

This is a prospective study of 182 women (38 yrs or younger) undergoing IVF-ET. Endometrial thickness, echo pattern and blood flow on transvaginal ultrasonography were recorded eight hours prior to hCG administration. The patients were divided into three groups: A (n = 10) with undetectable endometrial blood flow; B (n = 82) with sub-endometrial blood flow; C (n = 90) with both endometrial and sub-endometrial blood flow. According to IVF-ET outcomes, all patients were re-divided into three groups: 1 non-pregnancy (n = 92); 2 intrauterine pregnancy with live fetus (n = 70); 3 others (n = 20 including biochemical pregnancy, embryonic diapause, ectopic pregnancy and miscarriage). Intrauterine pregnancy with live fetus in Group C (62.2%) was much higher than that in Group A and B (0% and 17.1%, p less than or equal to 0.001). The implantation rate (33.2%) was much higher than that in Group A and B (0% and 19.90%, p less than or equal to 0.001). The pulsatility index, resistance index, and S/D of endometrial spiral arteries were 0.1 +/- 0.2, 0.6 +/- 0.1 and 2.5 +/- 0.4 in Group 2, which were much lower than those in Group 1 and Group 3 (p1-2 less than 0.001, p2-3 less than 0.05). The patients with detectable endometrial blood flow had higher clinical pregnancy rates and implantation rates.

## Background

In the menstrual cycle, the endometrium has no adhesive qualities until the implantation window phase, during which for a very short time, the endometrium allows the implantation of gestational sacs. This feature is referred to as endometrial receptivity [1]. Endometrial receptivity has, for a long time, been the major focus in the field of assisted reproduction because the synchronous changes of the endometrium with embryonic development is the basis for embryonic implantation.

Past studies related to endometrial receptivity were mainly focused on histopathological investigation of the endometrium, presented as endometrial dating by Noyes dating [2], or investigation of the receptors for estrogen, progesterone and other known factors related to endometrial receptivity. However, the diagnostic methods used for the above studies were invasive, hysteretic and a waste of time, and entailed intrauterine biopsy were not accepted by patients, because they were worried about subsequent miscarriage, and thus lost their value in guiding the assessment of endometrial receptivity, in those who wanted to be pregnant in this cycle.

With the advance of diagnostic ultrasonography, clinical use of ultrasonic technology has increased as a way to track changes in the uterine wall thickness, in the assessment of the developmental potential of the basal layer of the endometrium. With the increased resolving power and sensitivity of ultrasonography, more studies were conducted on the use of endometrial blood flow in predicting endometrial receptivity. To date, the advantages of ultrasonography including its non-invasiveness, repeatability, real-time monitoring and predictability, have gained more medical attention; however, ultrasonographic findings indicating changed endometrial receptivity have not yet been acknowledged. Therefore, in this prospective study, we aim to examine the value of ultrasongraphic technology in the assessment of endometrial and subendometrial blood flow and clarify its relationship with in-vitro fertilization and embryo transfer (IVF-ET) outcomes, in the clinical setting.

## Methods

### Patients

182 women, all under the age of 38, undertook IVF-ET at Peking University 3^rd ^Hospital, between November 1st and December 31th, 2008. Of them 110 had primary infertility and 72 had secondary infertility. Of the 182 patients, 100 had infertility caused by tubal disorders, 55 by male factors and 27 by ovulation failure. Serological tests showed the patients' FSH were lower than 15 IU/L. Ultrasonography demonstrated that the patients had uteri of normal size and shape. Every patient gave written informed consent voluntarily prior to participating in the study, which was approved by the Ethics Committee, Faculty of Medicine, Peking University Third Hospital. All patients received a long protocol of pituitary down regulation with Alarelin (a kind of GnRH agonist made by GL Biochem (Shanghai) Ltd, Shanghai, China) 150 mg by s.c. injections once daily from the midluteal phase of the cycle preceding the treatment cycle and received Gonal-F (a highly pure, recombinant form of human follicle stimulating hormone, made by EMD Serono, Inc., Darmstadt, Germany) for ovarian stimulation. HCG 10,000 IU was given i.m. when the leading follicle reached 18 mm in diameter and there were at least three follicles of more than 16 mm in diameter. Each patient was evaluated only once during the study period. Of the two to three embryos implanted, at least one was of good quality.

### Methods

Ultrasound measurement of all patients was performed by Dr. Wang Lina, 8 hours before hCG injection, after they had rested for at least 15 minutes and completely emptied their bladders. Endometrial thickness, pattern, volume and the blood flow of the uterine arteries, ovarian arteries and endometrial spiral artery were performed by transvaginal 6.5 MHz ultrasonography with Envisor C Color Doppler Ultrasound (Royal Philips Electronics Inc., Amsterdam, Holland).

The ultrasonic findings were classified according to the morphology of the endometrium [3]: Type A, triple-line or multi-layered type; Type B, slight-triple-line type; Type C, even-and-strong-echoed type. Using color Doppler in the two-dimensional mode, flow velocity waveforms were obtained from the ascending main branch of the uterine artery on the right and left side of the cervix before it entered the uterus in a longitudinal plane. Ovarian blood flow were obtained by color Doppler and the average of two ovaries were calculated. Endometrial blood flow was detected by intra-endometrial or the adjacent sub-endometrial regions within 10 mm of the echogenic endometrial borders. Double thickness of the endometrium was measured (maximum distance between each myometrial/endometrial interface through the longitudinal axis of the uterus). Thereafter, color Doppler energy was superimposed on the 2-D Doppler studies were performed on selected areas. The pulsatility index (PI) and resistance index (RI) of the uterine, ovary and endometrial arteries were calculated electronically when three similar, consecutive waveforms of good quality were obtained. Analysis was used together with computer algorithms to form the endometrial volume and indices of blood flow within the endometrium. The parameters were analyzed by software [4] for: (i) resistance index (RI): the difference between maximal systolic blood flow and minimal diastolic flow divided by the peak systolic flow (S-D/S); (ii) pulsatility index (PI): the difference between maximal systolic blood flow and minimal diastolic flow divided by the mean flow throughout the cycle (S - D/mean); (iii) the ratio between peak systolic flow and lowest diastolic flow (S/D). These three parameters express the resistance to flow from the point of measurement downstream. The patients were divided into three groups according to the condition of the endometrial blood flow [5]: In Group A, no endometrial blood flow was detected; Group B had sudendometrial blood flow detected, and Group C had both endometrial and subendometrial blood flow detected.

### Diagnosis of pregnancy

The patients were followed up for IVF-ET outcomes. On Day 14 after embryo transfer, blood hCG of all the patients were tested and on Day 21, the patients with seropositive results were given another blood hCG test. On Day 30, the pregnant patients received vaginal ultrasonography, in which the diagnosis of clinical pregnancy was established based on the type B ultrasonographic finding of an intrauterine gestational sac, or the existence of villus in abortus or chorionic tissue on pathological examination. The diagnosis of extrauterine pregnancy was confirmed by laparoscopy. Miscarriage was defined as pregnancy loss before 20 weeks of gestation. The patients were divided into three groups: Group 1--non-pregnant group, Group 2--intrauterine pregnancy with living fetus (displaying a gestational sac and primary embryonic pulse on day 30 by transvaginal ultrasonography), Group 3--poor pregnancy outcome, including the patients with biochemical pregnancy, ectopic pregnancy, or miscarriage.

Luteal support: All the patients received the same luteal support by progesterone since the day of embryo transfer in which progesterone 20 mg was given daily from the day of ET.

### Statistical analysis

Statistics Package for Social Sciences (SPSS) 13.0 software (SPSS Inc USA) was used to analyze the data. Continuous data are presented as mean +/- SD. The potential confounding effects of continuous variables related to pregnancy were assessed by analysis of variance (ANOVA) and Independent Samples T Test. Numeration data were assessed using the chi-square test. A P value of less than 0.05 was considered statistically significant.

## Results

All the patients (182) had color endometrial blood flow imaging on the day of hCG injection: 172 had visible endometrial or subendometrial blood flow. The detectability rate of endometrial blood flow was 94.5% (172/182 cases). The clinical pregnancy rate was 41.8% (76/182 cases). Of the 429 embryos transferred, 108 were implanted, thus the implantation rate was 25.2%, or 108/429 embryos.

### Comparison between pregnant patients and non-pregnant patients

No significant changes were observed in the comparison of Group 1, Group 2 and Group 3 in relation to BMI (kg/m^2^), causes of infertility, type and duration of infertility, number of follicle retrievals, embryos transferred, and changes between basal serological hormone levels and levels on the day of hCG administration (P greater than 0.05, Table. 1).

**Table 1:** Comparison between pregnancy group and non-pregnancy group

Group		Group 1 Non-pregnancy (n = 92)	Group 2 Intrauterine pregnancy with live fetus (n = 70)	Group 3 Poor pregnancy outcomes (n = 20)	*P *value
Age (y)		32.8 +/- 3.6	32.1 +/- 3.3	31.8 +/- 4.0	0.120

BMI (kg/m^2^)		21.7 +/- 2.5	22.1 +/- 2.6	21.5 +/- 2.9	0.350

Duration of infertility (y)		5.6 +/- 3.6	5.5 +/- 3.3	6.1 +/- 3.6	0.496

No of eggs collected		13.6 +/- 7.3	13.2 +/- 5.7	11.9 +/- 6.8	0.802

No. of embryos transferred		2.3 +/- 0.5	2.4 +/- 0.6	2.4 +/- 0.5	0.254

Causes of infertility	Obstruction of the oviduct	51	39	10	0.541
	
	Husbands' disorder	27	23	8	0.971
	
	Ovulation disorders	17	8	2	

Type of infertility	Primary	56	43	11	
	
	Secondary	36	28	8	0.16

Ovulation induction protocol	Long protocol	21	11	7	
	
	Short protocol	71	59	13	

Basal FSH (μIU/mL)		7.3 +/- 3.1	7.4 +/- 3.3	7.8 +/- 5.3	0.563

Basal E_2 _(pmol/L)		97.0 +/- 40.2	92.3 +/- 27.7	82.0 +/- 36.7	

E2 on the day of hCG administration (nmol/L)		6586.7 +/- 4229.8	6481.0 +/- 5288.1	6450.0 +/- 3563.4	0.998

P on the day of hCG administration (nmol/L)		2.6 +/- 1.8	2.2 +/- 1.6	2.3 +/- 1.0	0.178

### The relationship between endometrial blood flow and pregnancy outcomes

**Figure 1:**
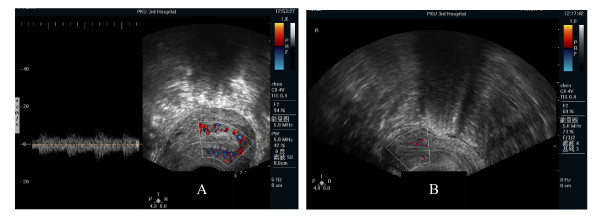
**Patients' outcome**. It has a good outcome for patients with abundant endometrial blood flow (A) of IVF-ET, and insufficient flow has a bad outcome (B).

In the three groups divided according to endometrial blood flow imaging, Group A, Group B and Group C, we found the intrauterine living fetus rates and embryo implantation rates of Group C significantly higher than those in Group A and Group B (Table. 2, Figure 1).

**Table 2:** The relationship between the blood flow of spiral arteries and IVF-ET outcomes

Groups	Pregnancy group (n, %)			Implantation rate of embryos	
	Non-pregnancy	Intrauturine living embryo	Poor pregnancy outcome	No. implanted embryos	%
Group A (n = 10)	9 (90%)	0 (0%)	1 (10%)	0 (0/23)	0
Group B (n = 82)	54 (65.9%)	14 (17.1%)	14 (17.1%)	40 (40/201)	19.9
Group C (n = 90)	29 (32.2%)		5 (5.6%)	68 (68/205)	33.2
x^2^, *P value*					
A-B		2.74, *0.254*			5.57, *0.018*
A-C		14.43, *0.001*			10.87, *0.000*
B-C		36.70, *0.000*			9.15, *0.003*

### The relationship between color Doppler imaging parameters and IVF-ET outcomes

**Figure 2:**
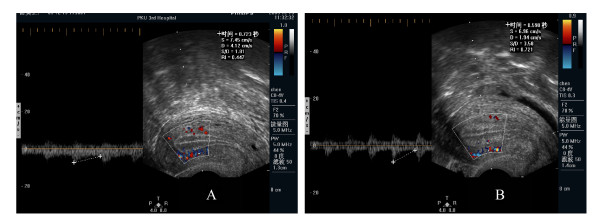
**Endometrial blood flow**. It has a better outcome for parameters with low endometrial blood flow (such as low PI, RI, S/D) (A) of IVF-ET compared with those who had high blood flow parameters (B).

We found no relationship between pregnancy outcomes and the color Doppler imaging parameters such as the S/D, PI and RI of uterine and ovarian artery blood flow, endometrial thickness, morphology and size of the ovaries on the day of hCG administration. But we noted that the S/D, PI and RI of endometrial blood flow in Group 2 (intrauterine living embryos group), were significantly lower than those in the other two groups (Table. 3, Figure 2).

**Table 3:** The relationship between parameters used in color Doppler imaging of blood flow and IVF-ET outcomes

Group	Endometrial		Uterine blood flow			Ovarian blood flow			Endometrial blood flow			Morphology of endometrium		
	thickness on the day of HCG injection (mm)	volume on the day of HCG injection (ml)	S/D	PI	RI	S/D	PI	RI	S/D	PI	RI	Type A	Type B	Type C
Group 1 (n = 92)	10.1 ± 1.9	7.4 ± 6.5	6.2 ± 4.2	2.0 ± 0.6	0.8 ± 0.1	2.6 ± 0.5	1.0 ± 0.3	0.6 ± 0.4	2.8 ± 0.4	1.11 ± 0.2	0.64 ± 0.1	65	26	1
Group 2 (n = 70)	10.5 ± 1.6	7.4 ± 2.3	5.4 ± 2.6	2.0 ± 1.1	0.8 ± 0.1	2.8 ± 0.9	1.1 ± 0.4	0.6 ± 0.1	2.5 ± 0.4	0.98 ± 0.2	0.60 ± 0.1	50	19	1
Group 3 (n = 20)	10.4 ± 1.8	7.5 ± 3.7	5.1 ± 1.4	1.9 ± 0.3	0.8 ± 0.1	2.6 ± 0.8	0.9 ± 0.2	0.6 ± 0.1	2.8 ± 0.4	0.98 ± 0.2	0.63 ± 0. 1	13	7	0
F, *P value*														
F1-2, *p*	-0.29, *0.607*	3.33, *0.563*	1.29, *0.052*	-2.72, *0.267*	10.20, *0.483*	-0.23, *0.152*	0.28, *0.558*	0.10, *0.339*	0.29, *0.000*	0.13, *0.000*	0.04, *0.000*	x^2 ^= 0.73, 0.948		
F2-3, *p*	-0.19, *0.834*	12.49, *0.180*	0.16, *0.883*	2.85, *0.467*	-40.17, *0.080*	0.04, *0.856*	0.35, *0.641*	0.004, *0.976*	-0.25, *0.013*	0.005, *0.933*	-0.03, *0.019*			
F1-3, *p*	-0.49, *0.591*	15.82, *0.084*	1.14, *0.278*	0.13, *0.973*	-29.98, *0.175*	-0.18, *0.441*	0.63, *0.388*	0.10, *0.492*	0.04, *0.689*	0.14, *0.026*	0.008, *0.593*			

### The value of color Doppler imaging in predicting IVF-ET outcomes

**Figure 3:**
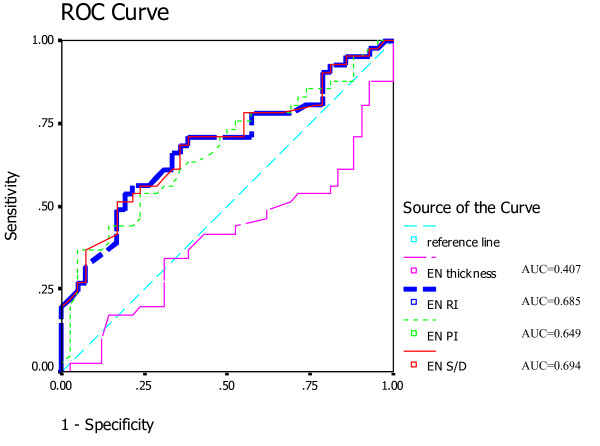
**ROC curve**. ROC curve of endometrial thickness and endometrial blood flow parameters (RI, PI, S/D).

In the ROC curve for comparison of sensitivity, 1-specificity, and positive and negative predictive values of the clinical pregnancy group and intrauterine living embryos group, we found the areas under the ROC curve of S/D, PI and RI of the endometrium were above the reference line (Figure 3), indicating correlation between blood flow parameters detected by colour Doppler imaging and IVF-ET outcomes (Table 4).

**Table 4:** Correlation between parameters (PI, RI, S/D) of endometrial blood flow and IVF-ET outcomes

Grouping	Sensitivity (%)		Specificity (%)		Positive Predicative Value		Negative Predicative Value	
	Clinical pregnancy group	Intrauterine living embryos group	Clinical pregnancy group	Intrauterine living embryos group	Clinical pregnancy group	Intrauterine living embryos group	Clinical pregnancy group	Intrauterine living embryos group
S/D > 3	60.5	65.3	83.3	79.4	57.4	45.7	58.7	79.3
PI > 1.2	66.3	67.3	88.1	88.2	56.4	45.1	69.4	79.6
RI > 0.7	61.0	67.3	83.3	79.4	56.5	45.0	68.6	78.4

## Discussion

In the field of assisted reproduction, transvaginal ultrasonography is often used to examine endometrial thickness, morphology and blood flow status to predict uterine receptivity [6]. However, many issues, such as time of ultrasonography examination and its value in predicting IVF-ET outcome remain uncertain.

The time of choice for transvaginal ultrasonography varied in the past studies. Much research was based on records of ultrasonographic blood flow after the day of hCG injection or on the day of embryo(s) transfer. Because of the reports on increased impedance of uterine arteries after hCG injection and its influence on the predictive value of ultrasonography [7], we believe conducting ultrasonography before hCG allows physicians to make decisions on the time of hCG injection or treatment to improve uterine artery blood flow [8]. Therefore, in our study, ultrasonography was conducted on the day of hCG injection.

Many researchers believe that an endometrial thickness during ovulation that is below a specific value will reduce the likelihood of pregnancy to almost zero. Past reports of this thickness ranged between 5 and 8 mm. The research by Issacs et al found the ideal range of endometrial thickness for implantation between 9 and 11 mm [9]. The relationship between endometrial morphology and IVF-ET outcomes is also uncertain. Jarvela et al [10] considered that endometrial thickness and morphology related to IVF outcomes. In their study on patients receiving gonadotropin injections in an IVF-ET cycle, 44.8% of the patients with a 3-line pattern before hCG injection became pregnant; and 80% of the patient with a 3-line pattern on the day of ovum collection became pregnant. However, research by Ng et al reported no relationship between endometrial thickness, morphology and pregnancy outcomes [11]. And in a recent study, endometrium pattern, endometrium thickness, and end-diastolic blood flow were shown to be the most effective combination for evaluation of uterine receptivity [12]. Our study ascertained that endometrial thickness and morphology had no relationship with IVF-ET outcomes, because in our study, those whose endometrium thickness was less than 7 mm were not accepted for transferring their embryos.

Early colour Doppler ultrasonography is mainly used to assess endometrial receptivity through measuring uterine blood flow. Our study, however, found no association between uterine arterial blood flow and pregnancy outcome, as describe by Engmann et al [13] and we suggest that uterine artery S/D, RI and PI could not be used alone to predict endometrial receptivity.

Does endometrial blood flow represent the receptivity of the endometrium? Related studies found that the border area between the myometrium and endometrum, a thin low-echo layer, plays an important role in endometrial receptivity [14]. The endometrial flow blood detectability rate was rather low in past years because of the limited detection technology of that time. Now, with the advance of ultrasonography, colour Doppler energy imaging has been used in endometrial blood flow assessment. Colour Doppler energy imaging is a technology based on the total integral of energy frequency spectrum. It visualizes blood flow with the energy of moving reflectors and enjoys the advantages of high sensitivity to slow blood flow, while being less dependent on angles and providing a less cluttered image.

The detection rate of endometrial blood flow in our study was 93.3%, similar to relevant reports [15]. The condition and existence of endometrial blood flow showed direct association with pregnancy outcomes and implantation rate. In this study, only one patient without endometrial blood flow had a biochemical pregnancy, and none of the patients without blood flow had clinical pregnancy. Detection rates of endometrial blood flow in the groups of embryo diapause, miscarriage and ectopic pregnancy were significantly lower than that in the intrauterine live fetus group, suggesting an association between the condition of endometrial blood flow and embryo implantation and development. Our findings also showed that the pregnancy rate and implantation rate of the patients with detected endometrial and subendometrial blood flow were significantly higher than those with only subendometrial blood flow detection or those without detected blood flow, while the miscarriage rate in the patients with detected endometrial and subendometrial blood flow was significantly lower. The endometrial thickness and the speed of uterine blood flow had no impact on pregnancy outcomes. These findings support the association between endometrial blood flow and endometrial receptivity.

In this prospective study on 182 patients undergoing IVF-ET, the data has good comparability. We found that patients with detected endometrial blood flow had a higher pregnancy and implantation rate. Therefore, blood supply and distribution of the endometrium had a strong association with the possibility for embryo implantation and development, indicating the value of detecting the existence of endometrial blood flow in predicting IVF-ET outcomes. Research [16] on increased pregnancy rates and better IVF-ET outcomes with improved endometrial blood flow support our points. In another study, endometrial volume and 3D power Doppler indices were shown to be statistically significant in predicting the cycle outcome when one grade 1 or no grade 1 embryos are transferred, and thus thought it could be helpful data in a single-embryo transfer policy [17]. As in our study, we also found a significant increasing multiple pregnancy rate in those who had better endometrial blood flow. And in a recent paper, Ng et al [18] discuss the relationship of endometrial blood flow between those who with a thin (less than or equal to 8 mm) endometrium and in those with a low volume (less than or equal to 2.5 ml) endometrium. It was found that 3D power Doppler flow indices of the endometrial and subendometrial regions were significantly lower in patients with a low volume endometrium compared with those with a normal volume endometrium. Endometrial and subendometrial vascularity measured by 3D power Doppler ultrasound was significantly lower (P less than or equal to 0.003) in patients with a low volume endometrium, but not in those with a thin endometrium. But the 3-D Doppler technique was not used in our study. It requires further study to explore the potential benefit of improving IVF-ET outcomes by intervening in circumstances of poor endometrial blood flow, or freezing embryos until endometrial blood flow is good enough for IVF-ET.

## Competing interests

The authors declare that they have no competing interests.

## Authors' contributions

WL carried out ultrasound examination, participated in the sequence data collection and analysis and drafted the manuscript. QJ conceived of the study, and participated in its design and coordination and helped to draft the manuscript. LR participated in the design of the study and performed the statistical analysis. ZX and LZ participated in the patients' selection. All authors read and approved the final manuscript.
